# Thermodynamic Coupling Forming Performance of Short Fiber-Reinforced PEEK by Additive Manufacturing

**DOI:** 10.3390/polym16131789

**Published:** 2024-06-25

**Authors:** Qili Sun, Xiaomu Wen, Guangzhong Yin, Zijian Jia, Xiaomei Yang

**Affiliations:** 1 College of Mechanical and Electrical Engineering, Nanjing University of Aeronautics and Astronautics, Nanjing 210016, China; 2 National Key Laboratory of Transient Impact, No.208 Research Institute of China Ordnance Industries, Beijing 102202, China; zjjr116@163.com; 3 Escuela Politécnica Superior, Universidad Francisco de Vitoria, Ctra. Pozuelo-Majadahonda Km 1.800, 28223 Madrid, Spain; amos.guangzhong@ufv.es; 4 Fibre and Particle Engineering, University of Oulu, P.O. Box 4300, FI-90014 Oulu, Finland

**Keywords:** FDM, PEEK/CF composite, warp deformation, thermodynamic coupling, forming quality

## Abstract

In this work, the PEEK/short carbon fiber (CF) composites were prepared, a new thermodynamic coupling (preheating and impact compaction) process of the FDM method is proposed, and the warp deformation mechanism was obtained by finite element simulation analysis. Results show that a new method could improve the forming quality of an FDM sample. The porosity of FDM samples of the PEEK/CF composite gradually decreased from 10.15% to 6.83% with the increase in impact temperature and frequency. However, the interlayer bonding performance was reduced from 16.9 MPa to 8.50 MPa, which was attributed to the influence of the printing layer height change from the printhead to the forming layer. To explain the above phenomenon, a thermodynamic coupling model was established and a relevant mechanism was analyzed to better understand the interlayer mechanical and porosity properties of PEEK/CF composites. The study reported here provides a reference for improving the forming quality of fabricated PEEK/CF composites by FDM.

## 1. Introduction

Polyether ether ketone (PEEK) is a semicrystalline thermoplastic polymer that is composed of repeating units containing one ketone bond and two ether bonds in its main chain structure [1]. Given its excellent comprehensive properties, such as wear resistance [2], fatigue resistance, and good biocompatibility [3], it has myriad applications in automotive and aerospace, medical equipment, and other industries [4]. Forming and manufacturing methods for fiber-reinforced PEEK-based composite materials are also attracting attention. PEEK has a glass transition temperature of 143 °C and a melting point of as high as 343 °C. Hence, the commonly used forming methods for this material are injection molding and compression molding. However, prototypes with complex structures still cannot be obtained through either approach. This situation has become a crucial problem that restricts the wide application of PEEK-based composites.

Therefore, many scholars have carried out research on FDM composite materials. Wang [5] systematically studied the influence of short carbon fibers (CFs) and orthogonal building orientation on the flexural properties of printed PEEK composites. They found that the addition of CF enhances the uniform nucleation of PEEK during 3D printing, decreases layer-to-layer bonding strength, and greatly changes the fracture mode. Li [6] reported the effects of the mechanical properties of PEEK printed through FDM and its composites on biocompatibility. Their experimental results confirmed that printed CFR-PEEK specimens have significantly improved mechanical properties compared with printed pure PEEK. Laboratory experiments clearly showed that no toxic substances are introduced during the FDM manufacturing of pure PEEK and CFR-PEEK. Arif [7] studied the multifunctional performance of PEEK composites reinforced with carbon nanotubes (CNTs) and graphene nanoplatelets (GNPs). They reported that the Young’s and storage moduli of these materials increased by 20% and 66% under 3 wt.% CNT loading, respectively, and by 23% and 72% under 5 wt.% GNP loading, respectively. Moreover, they demonstrated that the crystallization temperature and crystallinity degree of FFF-PEEK increase with the addition of carbon nanostructures [8]. Tian [9] proposed three methods to improve the interface performance. Firstly, to improve the wettability of the resin and the fiber, the solution impregnation technology was used to pretreat the carbon fiber, and a PA slurry layer was introduced onto the surface of the carbon fiber to improve the fiber surface and resin interface bonding performance [10]. Secondly, physical cleaning and chemical modification of the carbon fiber surface by plasma treatment can effectively improve the bonding properties of two materials with a large polarity difference [11]. The interlayer performance of the 3D-printed sample after plasma treatment is improved by 70%. The effect of laser power and printing speed on the extrusion of continuous carbon fiber-reinforced PEEK composites is as follows: with the increase in laser power, the temperature of the junction point increases rapidly, the temperature of the binding point reaches about 420 °C, and the thermal accumulation of the surface layer decreases with the increase in the printing speed; 120 mm/min is the fastest printing speed at the highest thermal accumulation temperature, the interlaminar property of PEEK/CF can reach 56 MPa [12]. Thirdly, the interlaminar bonding property of PEEK/CF can obviously be improved with the decrease in scanning distance and interlaminar thickness. When the fiber content reaches about 35 wt.%, the interlaminar shear strength can reach about 35 MPa [11].

However, most of the studies focused on the printing and forming mechanical properties of 3D-printed composites utilizing filaments [13], and few reported on the warp deformation and formed quality of PEEK-based composites fabricated through 3D printing. In the process of FDM printing, the temperature difference is large because the thermoplastic material changes from a molten state to a solid state, which easily causes stress concentration in the composite material system, leading to micro-cracks, interface delamination, warping deformation, and other defects of the composite sample. These seriously affect the quality and mechanical properties of the FDM composites [14,15]. At present, there are few literature reports on the influence of residual stress on the warping deformation mechanism of formed samples. In addition, in the forming process of FDM stacking layer by layer, each layer will be subjected to multiple high-temperature thermal cycles, resulting in a comprehensive superimposed heat effect, resulting in a temperature gradient in the space of the heated plane, resulting in the deformation of the formed parts due to uneven temperature, and further leading to problems such as difficult interlayer fusion and large differences in the mechanical properties of the composite materials. Therefore, it is urgent to study the new forming process of preheating-impact-printing in the FDM forming process to improve the physical fusion characteristics between the new forming layer and the old layer and to improve the forming quality of the sample.

Based on the above analysis, the warpage deformation of PEEK/CF composites were predicted by finite element analysis, and the warpage deformation factors were analyzed during the forming process. Then, a new forming process integrating preheating, impact, and printing was proposed, and the influence of this process on the mechanics and forming quality of the forming samples was studied, which provides an important reference for improving the performance of FDM printing samples.

## 2. Experimental Materials and Test Methods

### 2.1. Experimental Materials

PEEK has a glass transition temperature of 143 °C and a melting point of as high as 343 °C. PEEK (450 G) was supplied by VICTREX Company (Lancashire, UK). Chopped CF (WD-100 AW) with an average fiber length of 100 µm was purchased from Nanjing Weida Composite Material Co., Ltd. (Nanjing, China) Prior to use, the PEEK and CF were dried for at least 3 h at 150 °C.

PEEK and PEEK/CF composite filaments were supplied by Shanxi Jugao AM Co., Ltd. (Taiyuan, China) The CF contents of the PEEK/CF composites were approximately 10 wt.%. The diameters of the filaments for 3D printing were controlled to 1.75 ± 0.05 mm.

### 2.2. Test Equipment and Test Conditions

(1) Shear strength test

Instrument: WDW-1 universal material testing machine, Beijing University of Chemical Technology Testing and Analysis Center.

Test conditions: Interlayer shear strength analysis was performed in reference to the standard ISO 14130:1998 (fiber-reinforced plastic composite material short beam method to determine the interlayer shear strength). The sample size was 20 mm × 10 mm × 3 mm, and the loading speed was 5 mm/min. The span was 10 mm, the upper support radius was 5 mm, and the lower support radius was 2 mm. The formula used to calculate the interlaminar shear strength $\tau_{M}$ (MPa) is:

$$\tau_{M}=\frac{3F}{4bh}$$

where *F* is the maximum load (N), *b* is the sample width (mm), and *h* is the sample thickness (mm).

(2) Porosity test (mercury intrusion method)

Instrument: POREMASTER GT60 mercury prosimeter. Test data were obtained by the Beijing Center for Physical and Chemical Analysis.

Test conditions: room temperature, pressure range of 0.20–30,000 psi, and mercury injection time of 70 min.

### 2.3. Fabrication of 3D-Printed Specimens

**Figure 1:**
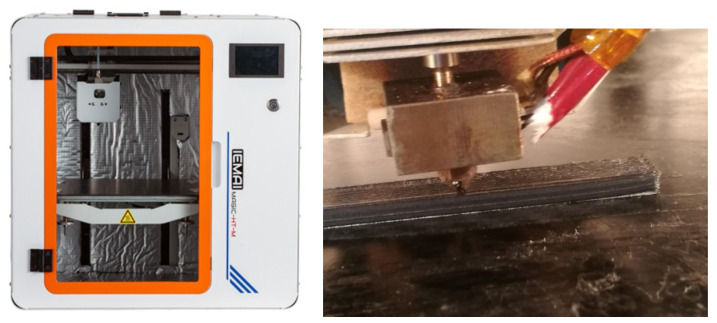
IEMAI 3D equipment and print sample process by FDM.

Five samples of each geometry were created by using PEEK/CF material to investigate the relationships among printing factors, mechanical properties, and porosity. The printing process is shown in Figure 1. The specifications of the geometric models of shear stress, GB/T1450.1-2005. The test sample models conforming to the relevant test standards were designed in Solidwork software 2020, and the geometric models were exported as files in stereolithography format for importation by the FDM software (Simplify 3D 3.0). The main FDM process parameters used to print the PEEK/CF composite samples are provided in Table 1.

### 2.4. Design of Multifunction Printhead

**Figure 2:**
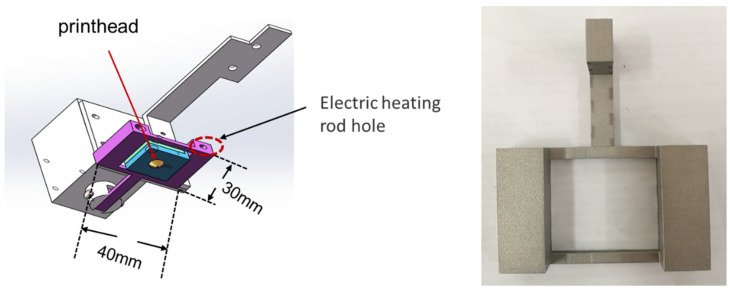
Preheating and shock device.

The multifunctional printhead is designed as a “fly swatter” structure and surrounds the printhead, as shown in Figure 2. The multifunction printhead includes the body printhead, impact compaction device, electric motor, and electromagnetic pulse controller. The device adopts an electric heating rod with a parallel structure and the corresponding temperature sensor, and the temperature of the electric heating rod is controlled by a micro-temperature controller to ensure the preheating effect of the impact device and uniform and stable preheating. The impact compaction device is used for the impact force size adjustment; an external digital dynamometer is also used for testing in the range of 0–5 N; the dynamometer is connected to the detection computer; the computer is utilized to operate the digital dynamometer through the software interface; and the test data are observed.

## 3. Analysis and Discussion of Experimental Results

### 3.1. Finite Element Simulation Analysis of Forming Warpage Deformation

#### 3.1.1. Hypothesis of Finite Element Model Analysis of FDM Process

The FDM process is a process in which PEEK wire is fused and extruded by a nozzle. During the printing process, the nozzle moves on the surface of the workpiece, the temperature gradient between the wire and the environment is large, and the heat source is concentrated. As a result, the temperature field is unsteady, and heat conduction and convection are the main heat transfer methods.

According to the abovementioned forming process characteristics, the numerical simulation model is established under the following assumptions:

(1) The material continuity assumption is that the material is treated with continuous heat transfer throughout the forming process.

(2) The model adopts a 3D finite element model for transient thermal analysis because the temperature field is unsteady.

(3) According to the printing process of a specific path, the “birth and death unit” method can be used for simulation, which activates regional units and loads heat sources one by one, according to the scanning speed of the device.

(4) The process of FDM has a problem of latent heat-of-phase transition in material forming, which is treated by the equivalent thermal melting method.

(5) The initial temperature of the unit is the temperature of the nozzle extrusion, and the convection heat transfer is conducted with the air after extrusion.

#### 3.1.2. Heat Source Model Analysis

According to the molding process of the 3D printer, the silk is heated by the nozzle from a solid state to a molten state. The nozzle is printed according to a specific path. Thus, the nozzle can be regarded as a moving heat source.

At present, heat source models can mainly be divided into the double-ellipsoid heat source model and the Gaussian heat source model. For the preparation process of molten deposition, the heat of the nozzle in the accumulation molding process is relatively concentrated, but no penetration effect occurs. The same position will also have differences with the change in time, and a large temperature gradient change will occur in the spatial position. This characteristic corresponds to the Gaussian heat source model. Thus, the Gaussian heat source model is used to simulate the nozzle heat source.

**Figure 3:**
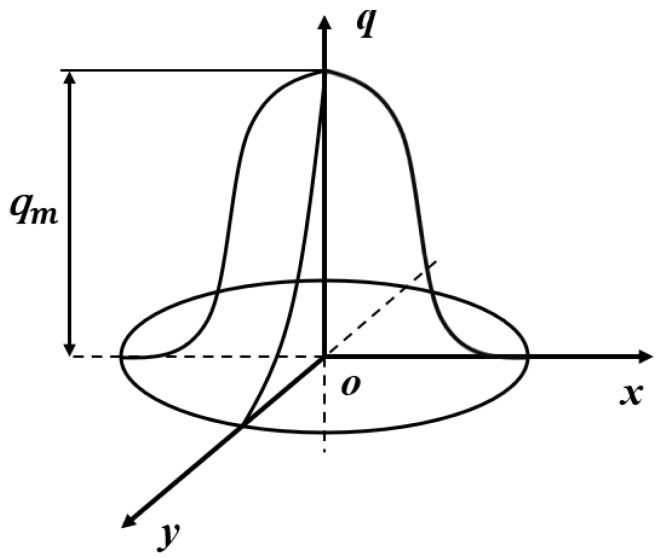
Heat flux distribution of the Gaussian heat source [16].

The heat source model of the Gaussian function distribution is shown in Figure 3 below:

The heat flux density distribution on the heating spot adopts the Gaussian mathematical model, and its heat flux density distribution function is as follows [17]:

(1) $$q\left(r\right)=\frac{3\eta P}{\pi R^{2}}\times \exp(-\frac{3r^{2}}{R^{2}})$$

where *R* is the effective heating radius of the heat source, *r* is the distance from any point on the heating surface to the center of the arc heating half, *η* is the effective power coefficient that truly acts on the molded parts after environmental effects such as convection and heat exchange, and *P* is the rated power of the heat source.

**Figure 4:**
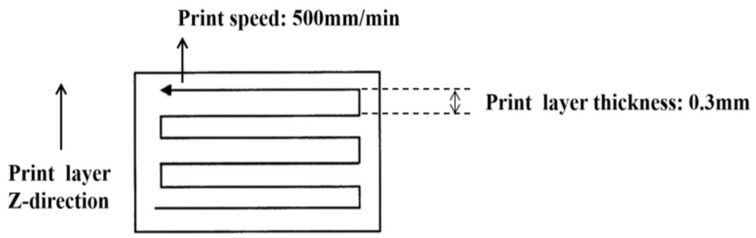
Trajectory of heat source movement.

According to the discussion above, the heat of the nozzle is relatively concentrated, the heat source of the printing process is the moving Gaussian surface heat source, and its moving trajectory is the printing path. Figure 4 shows the trajectory of heat source movement.

#### 3.1.3. Simulation Analysis of Warpage Deformation of Printed Samples

(1) Finite element model

① Model of pure PEEK composite

**Figure 5:**
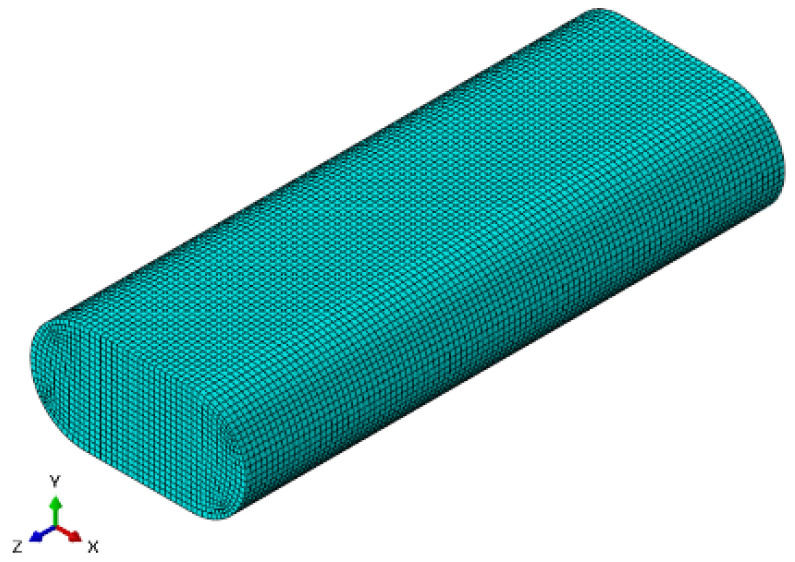
Model of pure PEEK composite.

The model uses the printing unit microsegment as the analysis object, and the structure is shown in Figure 5. The model length is 0.5 mm × 0.2 mm × 0.1 mm, and it contains 87,567 nodes and 81,400 units.

② Model of fiber-reinforced PEEK composite

**Figure 6:**
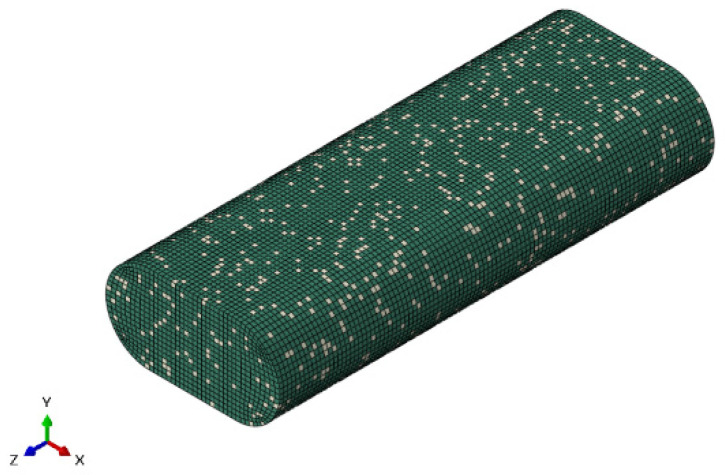
Model of PEEK/CF composite.

PEEK/short carbon fiber (CF) is a composite material reinforced with 10% chopped carbon fiber with a mixed volume fraction. The staple fiber is a strong anisotropic material, and the staple fiber is randomly mixed in the PEEK material. The model selects to print 5 mm microsegments as the analysis object. First, the discrete random algorithm is applied to discretize the short fibers into the model. Then, each short fiber is given random spatial direction in the model. Finally, the model is integrated for analysis. The model is shown in Figure 6.

(2) Simulation of printing layer sample

**Figure 7:**
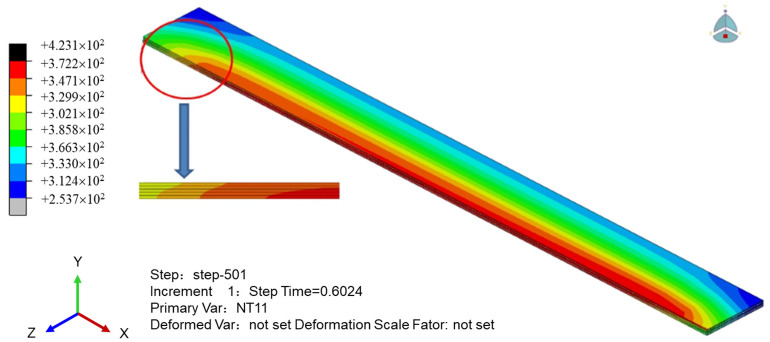
Temperature field cloud at the end of printing.

The five-layer sample is used as the analysis object to simulate the forming process of the multilayer sample; the heat source model is brought into the printing process simulation; the printing multilayer simulation method is like the single layer; the “life and death” unit technology is also used to simulate the printing process; and the unit “life and death” simulation path is consistent with the printing path. The heat flux density and temperature distribution cloud of the printed sample at a certain time are shown in Figure 7.

Figure 6 shows that the temperature of the sample during the printing process is the maximum near the printing terminus, and it diffuses radially to the periphery, which indicates a temperature gradient situation. During the printing process, the sample will absorb part of the heat due to the convection heat dissipation on the upper surface. As a result, the overall temperature of the sample tends toward the thickness direction of the ladder shape, and a trend of decreasing layer by layer is observed.

**Figure 8:**
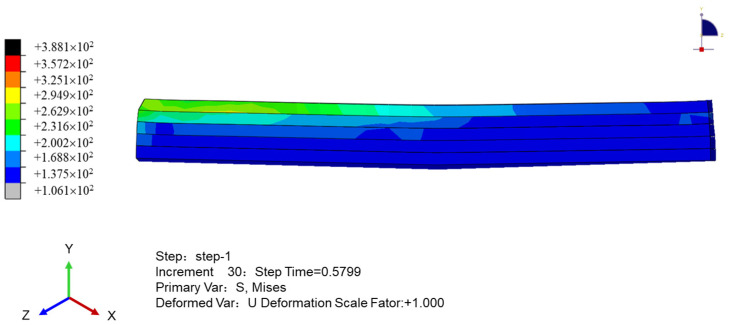
Sample stress cloud when printing is completed.

**Figure 9:**
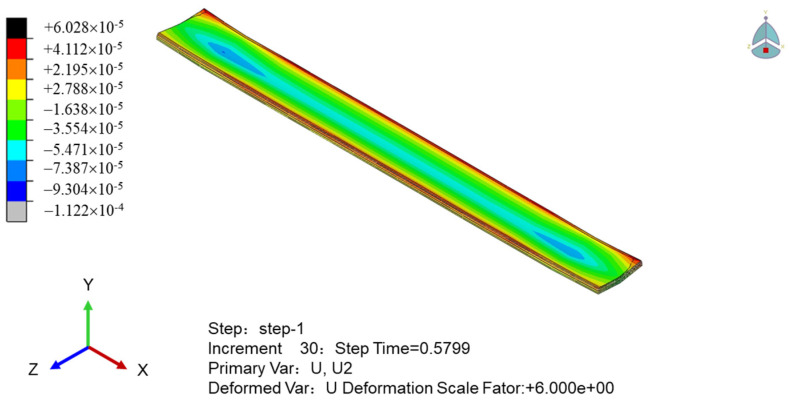
Pattern of sample thickness direction displacement cloud when printing is completed.

To obtain the relationship between the internal stress and temperature of the printed sample, we simulate the stress cloud and the displacement cloud in the thickness direction of the sample, as shown in Figure 8 and Figure 9, respectively. Figure 8 shows that the stress of the sample near the printing end lane is minimal; it decreases to the perimeter of the printing termination, and the overall stress field is an inverse wave layer. As shown in Figure 9, the stress of the sample is affected by the temperature field. The temperature of the upper surface of the sample drops sharply, the stress gradually increases, and the upper part of the sample shrinks with the increase in time. Meanwhile, the middle and bottom surface of the sample have heat conduction due to the printing process, the temperature drops slowly, and the internal stress changes slowly with the temperature. In general, the warpage around the upper part of the molded part changes the most, and the deformation of the sample is concave.

**Figure 10:**
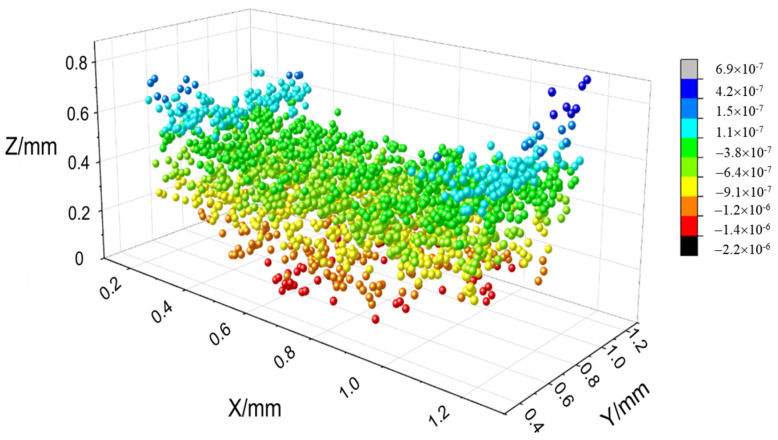
Calculation results of the stress field of PEEK/CF composites.

The stress field value of the PEEK/CF composite is obtained by extracting the nodal displacement of the bottom surface in Figure 10, to obtain the intuitive warpage deformation characteristics of the sample. The figure shows that the fiber-reinforced PEEK material is scattered with the discreteness of the spatial position of the chopped fiber due to its mixing characteristics. The overall microsegment stress is uneven, and the interface stress between the fiber and PEEK material is greater and shows stress concentration. The two stress field distributions caused by material and temperature have different characteristics of bottom warpage, and the fiber-reinforced PEEK material with a large internal stress has a large amount of warpage deformation.

**Figure 11:**
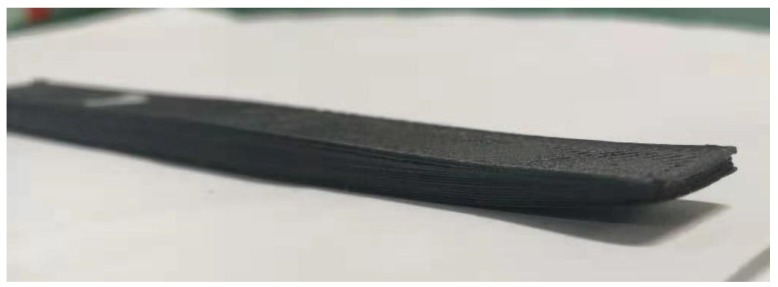
Printed samples (warpage deformation).

Figure 11 shows the actual printing situation, and the warpage deformation trend of the simulated printing results is consistent with the actual situation.

### 3.2. Integrated Forming Process of Preheating, Printing, and Impact Compaction

Previous studies have shown that the heat treatment temperature is higher than the glass transition temperature of PEEK thermoplastic material, PEEK shows high elastic characteristics, and the molecular chain segment has strong movement ability, which can effectively release the residual stress originally “sealing” in the matrix [18]. Therefore, the heat treatment process can reduce the maximum residual stress value of the fused deposition-forming sample, and the forming process of the PEEK/CF composite material needs to be heat treated. Therefore, we designed a multifunctional printhead to realize the forming process of preheating, printing, and impact compacting and expect to achieve the integrated forming of preheating, printing, and impact for improving the forming quality of the sample.

#### 3.2.1. Interlaminar Mechanical Properties of PEEK/CF Composites

(1) Influence of preheating temperature on the mechanical properties of interlayers

**Figure 12:**
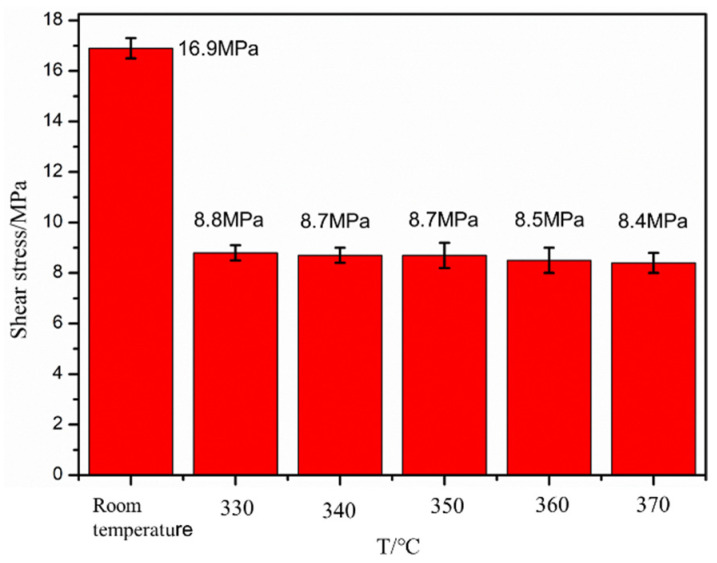
Effect of preheating temperature on shear strength.

In this section, the temperatures of the design hammer are 330, 340, 350, 360, and 370 °C, the impact frequency is 6 Hz, and the impact strength is 0.3 N. The influence of different preheating temperatures on the interlayer shear strength is studied, and the results are shown in Figure 12.

Figure 12 shows that the interlayer shear strength of the blank sample is 16.9 MPa; the interlaminar shear strength of the impact sample is the same, with a value of approximately 8.5 MPa; and the interlaminar strength of the PEEK/CF composite sample prepared through FDM after impact is reduced. This result may be due to two reasons. One is that the thermal radiation temperature of the impact hammer is low. The impact effect is a short thermal contact, and the heat flow is transmitted to the resin surface for a short time, which causes the “physical cross-linking point” between the hanging molecular chains in the layers of PEEK thermoplastic materials to become smaller; hence, the physical cross-linking effect between the layers is weakened, which, in turn, leads to a decrease in the bonding strength between the layers. The other reason is that the layer height of the printed sample increases after the impact action. The height of the extruded resin layer is significantly reduced due to the impact effect, but the spacing from the printhead to the printing layer increases, and the preprinted layer height of the next layer is larger when the impact strength is higher. As observed from the printing background, the interlayer shear strength is smaller when the layer height is larger. The sample after the impact does not achieve the desired results because of these two reasons.

**Figure 13:**
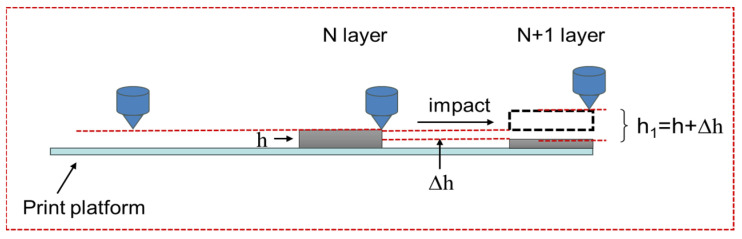
Chart of change in printing layer height.

This study establishes a model for the influence of the height change from the printhead to the forming layer on the interlayer performance, as shown in Figure 13, to understand the reasons for the degradation of the interlayer performance of samples after impact. For the design printing process parameters, the printing layer height is *h*. After the impact, the layer height thickness is reduced Δh. When the next layer is printed, the originally designed layer height *h* now becomes $h_{1}(h+\Delta h)$. The printing layer height increases. Thus, the interlayer shear strength decreases.

**Figure 14:**
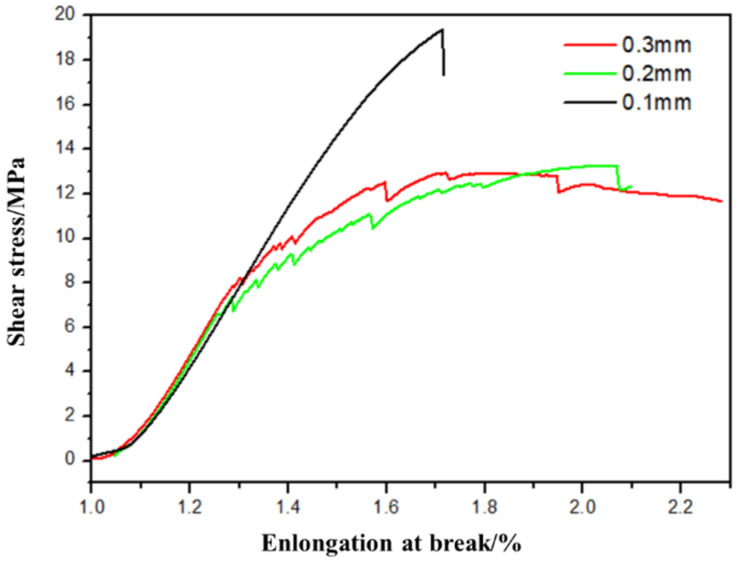
Effect of printing layer height on the shear strength of PEEK/CF composites.

A comparative experiment is designed in this section to study the influence of printing layer height on the interlayer performance of additive manufacturing of PEEK/CF composites for verifying the effect of the change in printing layer height on interlayer performance. Different layer heights of 0.1, 0.2, and 0.3 mm of FDM are designed, and the results are shown in Figure 14.

The figure shows that the interlayer shear strength of PEEK/CF composite prepared through additive manufacturing gradually decreases with the increase in printing layer height. The interlayer shear strength of PEEK/CF composite prepared by FDM is 19.37 MPa when the layer height is 0.1 mm; however, its interlayer shear strength is greatly reduced to 11.20 MPa when the layer height is increased to 0.3 mm. The reason is that the printhead has a compacting function for the molded part, and the lower printing layer height can improve the interlayer performance. Therefore, the printing layer height is conducive to improving the interlayer performance of the formed sample. At the same time, ensuring the impact effect and appropriately designing the layer height compensation mechanism are particularly important for improving the interlayer performance.

(2) Influence of impact strength and frequency on interlayer mechanical properties

**Figure 15:**
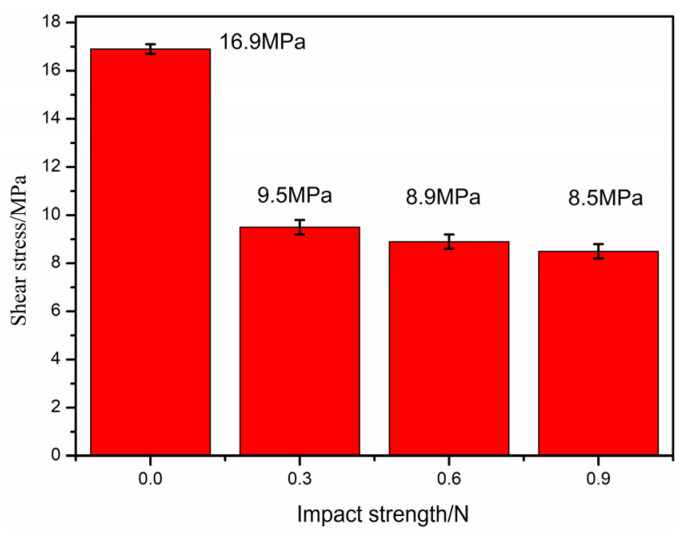
Effect of impact strength on interlaminar shear strength.

The real-time impact on the printing layer improves the quality of the formed sample. Preliminary studies have shown that the impact head will drive the printer to vibrate when the impact strength is higher than 1 N, and this condition is not conducive to printing. Therefore, the impact strength of this section is designed to be lower than 1 N (0, 0.3, 0.6, and 0.9 N), the preheating temperature and impact frequency are 350 °C and 6 Hz, respectively, and the influence of different impact strengths on the interlayer shear strength is studied, as shown in Figure 15, which shows that the interlayer shear strength of the blank sample is 16.9 MPa, and the interlayer shear strength of the sample after impact is lower than that of the blank sample. Compared with samples without any impact action, the impact strength also has a weakening effect on PEEK/CF composite samples prepared through FDM.

**Figure 16:**
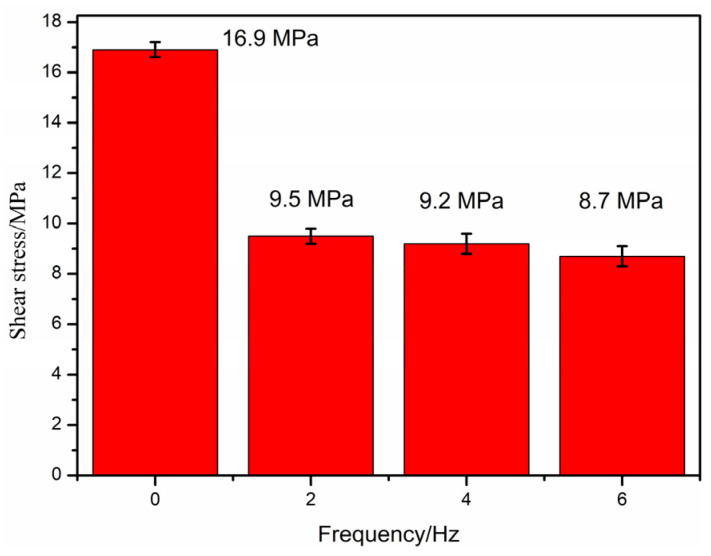
Effect of impact frequency on interlaminar shear strength.

Different impact frequencies (0, 2, 4, and 6 Hz) and preheating temperatures and impact strengths of 350 °C and 0.3 N, respectively, are designed in this section to study the influence of different impact frequencies on the interlaminar shear strength, as shown in Figure 16, for exploring the influence of the impact frequency on the interlaminar performance. The figure shows that the interlayer shear strength of the blank sample is 16.9 MPa, and the interlayer shear strength of the sample after impact is lower than that of the blank sample.

For the cause analysis, compared with the sample without any impact effect, the impact frequency also has the effect of weakening the interlayer of the PEEK/CF composite samples prepared through FDM. The reason is still the increase in the height between layers and the low preheating contact temperature, which result in weak interlayer performance.

#### 3.2.2. Porosity Analysis of Formed Samples under Thermodynamic Coupling

**Figure 17:**
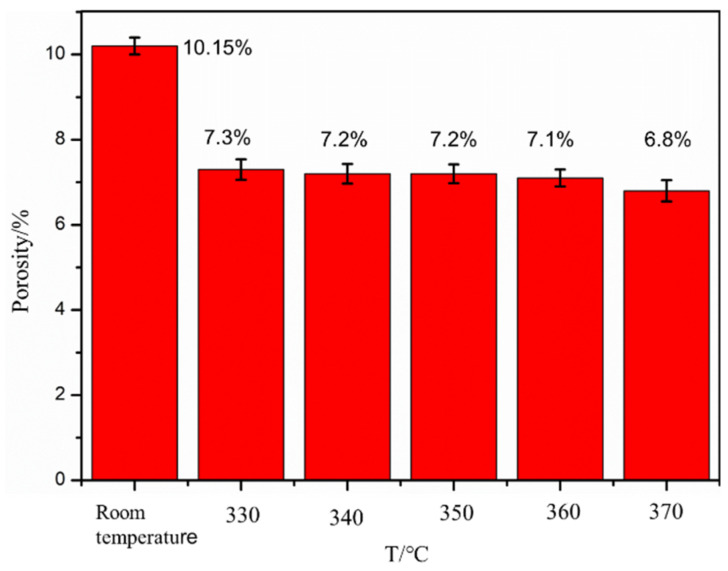
Effect of preheating temperature on porosity.

**Figure 18:**
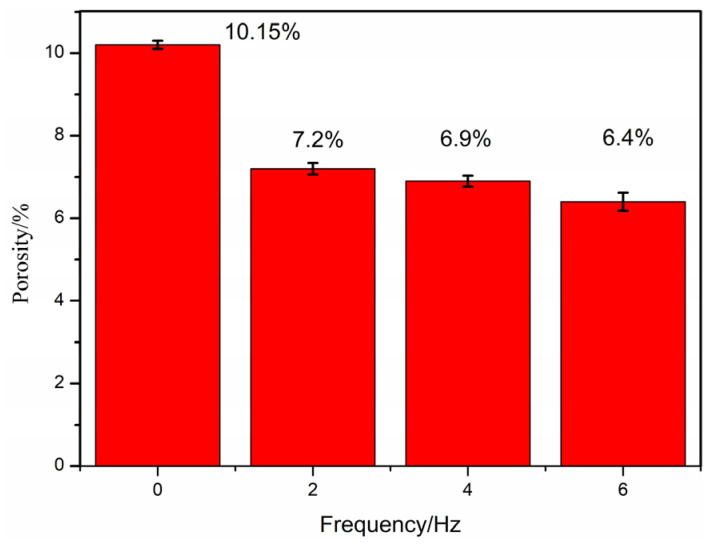
Effect of impact frequency on porosity.

The porosity of formed samples under different preheating temperatures and impact frequencies, as shown in Figure 17 and Figure 18, is studied in this section for investigating the porosity of FDM samples of PEEK/CF composites under thermodynamic coupling.

The influence of the preheating temperature on the porosity of PEEK/CF composites is studied when the design impact strength is 0.3 N and the impact frequency is 6 Hz. The influence of different preheating temperatures on the porosity of the formed samples is obtained. The results are shown in Figure 18. The figure shows that the porosity of the FDM samples of the PEEK/CF composite gradually decreases from 10.15% to 6.83% with the increase in preheating temperature. This result is due to the fact that the molecular segment movement of PEEK resin is more violent when the temperature is higher, and the gaps between the layers and the porosity of the material are lower when it is easier to compact under the action of an impact hammer. Therefore, preheating treatment during the printing process can reduce the porosity of the formed sample of the PEEK/CF composite.

The influence of different impact frequencies on porosity is studied with a preheating temperature of 350 °C and an impact strength of 0.3 N. The test results are shown in Figure 18. The figure shows that the porosity of the fused sedimentary samples of PEEK/CF composites (from 10.15% to 6.67%) gradually decreases with the increase in impact frequency. This result is due to the fact that the preheating temperature is constant, the PEEK/CF composite is easier to compact, and the porosity of its interlayer gaps and materials is lower when the impact hammer acts on the freshly printed sample more often. Therefore, the high impact frequency can reduce the porosity of the formed samples of PEEK/CF composites.

### 3.3. Mechanism of Thermodynamic Coupling

**Figure 19:**
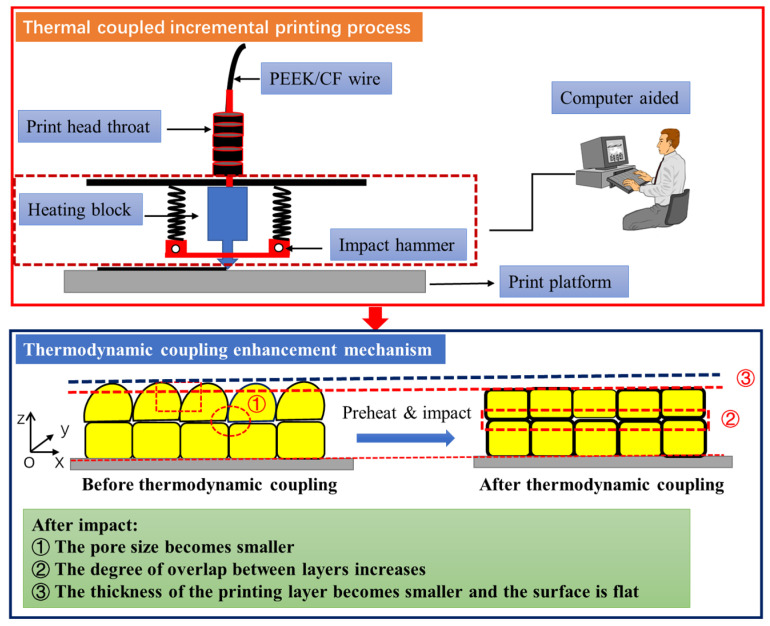
Thermodynamic coupling model.

In the process of additive manufacturing of parts, the remelting process of the print nozzle is the consolidation process of rapid melting and solidification of resin, which contains complex phenomena affected by multiple physical factors, such as heat transfer, mass transfer, diffusion, and phase change. A thermodynamic coupling model of PEEK/CF by FDM is established and its thermodynamic coupling mechanism is analyzed to better understand the interlayer mechanical properties of PEEK/CF composites under thermodynamic coupling. The thermodynamic coupling mechanism is shown in Figure 19.

FDM wire is a fiber-reinforced PEEK thermoplastic resin matrix composite material. In the forming process, the resin is melted, and after passing through the print head, it is fused, expanded, and then bonded onto the print layer. The macro-morphology shows a steamed bun shape, and the bond between layers is insufficient, resulting in higher interlayer porosity and a larger interlayer pore size. The high porosity ratio and large pore structure ultimately affect the interlayer mechanical properties. Therefore, in order to reduce the porosity and pore structure of the FDM forming process, a compact device similar to a “fly swatter” was designed, and a new forming process integrating preheating, printing, and compaction was proposed. The process is divided into three steps: the first is the preheating process, before printing the new forming layer, the forming layer to be covered, and preheating treatment (the temperature of the forming layer covered is generally about 100 °C printing chamber temperature, and the melting extrusion temperature of printing PEEK is about 340 °C), which can improve the melting bonding degree of the new printing layer and the old layer. The printing process is normally printed and overlaid on the “old layer”. During the impact process, after the “old and new layers” to be printed are fused, the impact of the physical action on the new and old layers can better promote interlayer adhesion and reduce the porosity and pore size.

After preheating and impact, the FDM samples of PEEK/CF composites undergo three changes. First, the porosity of the composite material is reduced. Second, the overlap of interlayer bonding increases. Third, the thickness of the sample printing layer is reduced.

**Figure 20:**
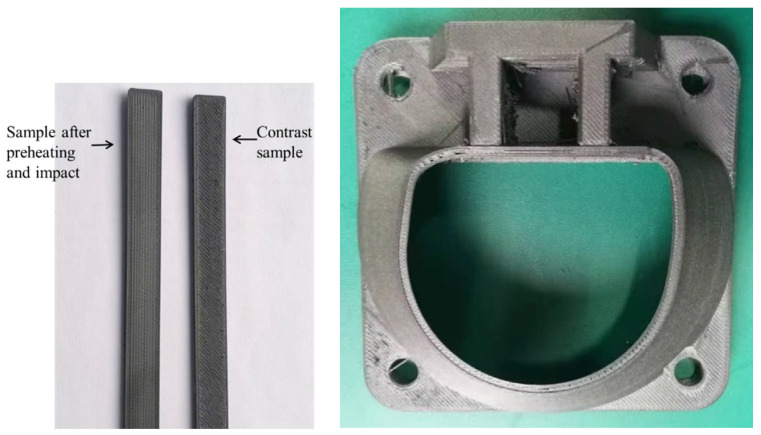
PEEK/CF composite before and after preheating and impact.

The system integration of a 3D printer based on a preheating and stamping device is completed, and a 3D printing experiment of carbon fiber-reinforced PEEK composite is conducted. The sample printing before and after preheating and shock is obtained as follows: Figure 20 shows that the sample without impact has a rough surface, and the surface has obvious ravines “plowed” by the printhead. After preheating and impact, the surface of the sample is relatively neat, which shows that the surface quality of the formed sample can be effectively improved by the preheating and impact process.

## 4. Conclusions

In this paper, PEEK/CF composites with 10% fiber content were prepared by FDM, and the following conclusions were obtained through comparative analysis and experimental testing:

(1) Finite element analysis shows that uneven heating and cooling and volume shrinkage caused by material phase change in the process of printing result in internal stress in the forming process of the printed sample, which leads to residual stress and deformation of the sample after forming. The fiber affects the stress field distribution of PEEK/CF composites and is affected by the material characteristics and temperature, and the warpage deformation is greater when the interface stress between the fiber and PEEK is greater.

(2) The printing verification of typical samples was conducted, which could effectively improve the printing performance and forming quality. The results show that the 3D-printed sample of carbon fiber-reinforced PEEK composite material printed without pressure has a rough surface. The surface of the sample has obvious ravines “plowed” by the printhead, and its surface is relatively neat after preheating and impact. The porosity study shows that the preheating and impact effects can reduce the porosity of the formed sample from 10.15% to 6.83%. The new process of preheating shock coupling to improve the forming quality of FDM is proposed, which provides a new method for improving the porosity and mechanical properties of FDM forming samples in the future.

(3) The interlayer properties of formed samples of PEEK/CF composite under thermal coupling were investigated. The results show that the interlayer properties of PEEK/CF composites decrease from 16.9 MPa to 8.9 MPa after thermal coupling because the height of the printing layer is increased after thermal coupling, which reduces the interlayer performance.

## Figures and Tables

**Table 1:** Printing process parameters.

Parameter	Value
Print head diameter/mm	0.4
Print head temperature/°C	400
Base plate temperature/°C	100
Print speed/(mm/min)	500
Print width/mm	0.5
Layer thickness/mm	0.3

## Data Availability

Data are contains in the article.
